# Relationship between *Helicobacter pylori* infection and white matter lesions in patients with migraine

**DOI:** 10.1186/s12883-022-02715-0

**Published:** 2022-05-21

**Authors:** Serkan Öcal, Ruhsen Öcal, Nuretdin Suna

**Affiliations:** 1 Department of Gastroenterology, University of Health Sciences Antalya Training and Research Hospital, Antalya, Turkey; 2 Antalya Training and Research Hospital Department of Neurology, Antalya, Turkey; 3 Department of Gastroenterology, Faculty of Medicine, Başkent University, Ankara, Turkey

**Keywords:** *Helicobacter pylori*, Migraine, White matter lesions

## Abstract

**Background/aim:**

White matter lesions (WML) are more frequently observed in migraine patients than in the average population. Associations between *Helicobacter pylori* (*H. pylori*) infection and different extraintestinal pathologies have been identified. Here, we aimed to investigate the association between *H. pylori* infection and WML in patients diagnosed with episodic migraine.

**Materials and methods:**

A retrospective study was conducted with 526 subjects with a diagnosis of episodic migraine. Hyperintensity of WML had been previously evaluated in these patients with brain magnetic resonance imaging (MRI) examinations. Previous endoscopic gastric biopsy histopathological examination of the same patients and reports on *H. pylori* findings were recorded. The demographic characteristics of the patients, such as age, gender and chronic systemic diseases such as hypertension and diabetes mellitus (DM) were recorded. Statistical evaluation was made.

**Results:**

Evaluation was made among 526 migraine patients who met the inclusion criteria, comprising 397 (75.5%) females and 129 (24.5%) males with a mean age of 45.57 ± 13.46 years (range, 18–69 years). WML was detected on brain MRI in 178 (33.8%) patients who were also positive for *H. pylori* (*p* <  0.05). Subjects who are *H. pylori*-positive with migraine, WML were observed at a 2.5-fold higher incidence on brain MRI (odds ratio: 2.562, 95% CI 1.784–3.680). WML was found to be more significant in patients with hypertension and migraine than those without (*p* <  0.001). Older age was also found to be associated with WML (OR = 1.07, 95% CI: 0.01–0.04, *p* <  0.001). The age (*p* <  0.001), *H. pylori* (*p* <  0.001), hypertension (*p* <  0.001), and hypertension + DM (*p* <  0.05), had significant associations in predicting WML according to the multivariate logistic regression analysis. The presence of hypertension had a higher odds ratio value than the other variables.

**Conclusion:**

It was concluded that *H. pylori* infection, as a chronic infection, can be considered a risk factor in developing WML in subjects with migraine.

**Supplementary Information:**

The online version contains supplementary material available at 10.1186/s12883-022-02715-0.

## Introduction

Migraine is a multifactorial and neurovascular condition characterized by recurrent headache episodes accompanied by autonomic nervous system disorder [1, 2]. One-third of migraine patients have an aura. Migraine affects over 17% of women and 5–8% of men [2]. Although the exact pathophysiology of migraine has not been elucidated as yet, in patients with a genetic predisposition, exogenous and endogenous stimuli may trigger pain attacks [3]. Various mechanisms, including pain mediators such as calcitonin gene-related peptides, and neurotransmitters such as serotonin, are currently discussed in the physiopathology of migraine [4].

*Helicobacter pylori* (*H. pylori*) is a gram-negative, microaerophilic, spiral-shaped and flagellated bacterium [5]. H.pylori is responsible for infection, chronic active gastroenteritis, duodenal and gastric ulcer and even stomach cancer [6]. Relationship between H.pylori infection and heart diseases such as coronary heart disease, vascular diseases such as primary Raynaud’s phenomenon, neurological diseases such as migraine, Alzheimer’s disease, and mild cognitive impairment, and numerous extraintestinal conditions such as iron deficiency anemia have been recently studied and discussed [7–9]. However, no consensus has been reached, and the results remain controversial.

Recently, many studies investigating the effect of *H. pylori* infection on migraine physiopathology have been published [6, 10]. The role of *H. pylori* in the pathogenesis of migraine has been suggested based on the relationship between the host immune response to bacteria and the extended-release of vasoactive substances [11–14]. The inflammatory response to *H. pylori* infection in humans comprises infiltration of lymphocytes, neutrophils, and monocytes, into the gastric mucosa and submucosa [15, 16]. The activation and aggregation of immune cells in the gastric mucosa and submucosa includes epithelial-derived chemotactic peptides (chemokines), such as interleukin IL-8 and growth-regulated oncogene (GRO) -α, bacterial chemotaxis, and proinflammatory cytokines released by mononuclear phagocytes, such as tumor necrosis factor-alpha (TNF-α), IL-1, and IL-8 [16, 17]. Vasoactive substances and psychological or physical stress that can determine the clinical signs and symptoms of the disease, special foods, and sex hormones are among the well-known trigger factors that may induce cerebral vascular hyper-reactivity [14, 18].

Migraine has been identified as an independent risk factor for hyperintense lesions seen in the white matter in cerebral magnetic resonance imaging (MRI) [19]. Nonspecific white matter lesions (WML) appear as hyperintense lesions in T2-weighted (T2W) or fluid-attenuated inversion recovery (FLAIR) sequences. The main pathological features of WML are demyelination, pale myelin, vacuolization, and oligodendrocyte apoptosis [20]. There are many studies focusing on the anatomy, cerebral blood flow autoregulation, blood-brain barrier disruption, venous collagenosis and genetic factors of WML to understand its pathogenesis [20, 21]. In this study, we investigated *H. pylori* infection as a risk factor for WML in patients with migraine and evaluated their relationship.

## Materials and methods

### Study

This study was a retrospective analysis of *H. pylori* infection in patients with episodic migraine. Approval for this retrospective study (decision no: KA16 / 188) was accepted by Baskent University Ethics Committee. The study was designed according to the principles of the Helsinki Declaration. The written informed consent from the patients could not be taken due to the study’s retrospective design and the unanimity of data.

### Patients

**Fig. 1:**
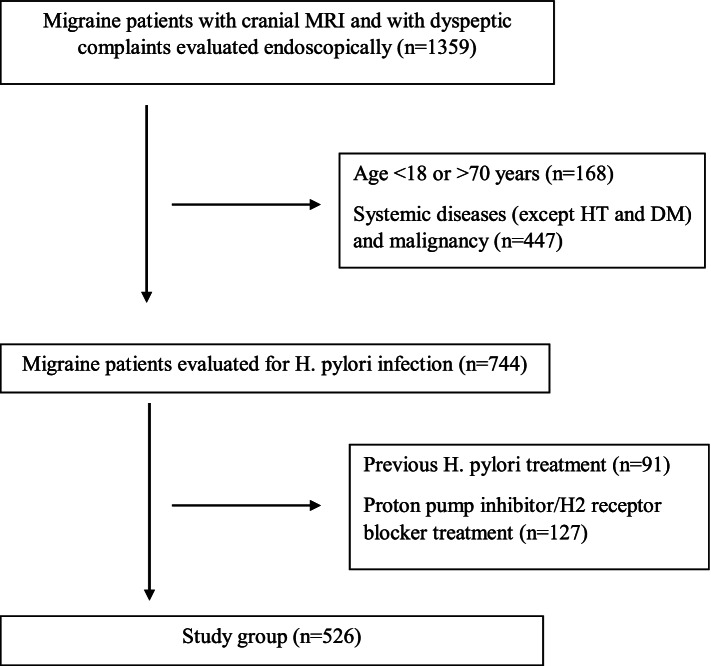
The flowchart of the study

The files were examined of patients who presented at Baskent University Ankara Hospital Neurology Outpatient Clinic between 2015 and 2019 and were evaluated by a neurologist and diagnosed with episodic migraine according to the International Classification of Headache Disorders, 2nd edition (ICHD-II) [22]. Those patients with a previous cranial MRI were included. Patients diagnosed with other systemic diseases such as kidney, heart, endocrine, immunological, a history of malignancy, or < 18 or > 70 years were excluded. Of the patients with dyspeptic complaints (upper abdominal pain-discomfort-distension, retching, postprandial fullness, nausea, belching, vomiting) examined in the gastroenterology outpatient clinic, the study included those who were evaluated for *H. pylori* in endoscopic histopathological examination. Among these, the patients who received *H. pylori* infection treatment or used proton pump inhibitor or H2 receptor blocker drugs within the past 2 weeks were omitted. A total of 526 migraine patients meeting these criteria were enrolled (Fig. 1).

### Interventions

#### Cerebral magnetic resonance imaging

The radiological evaluations were made from cranial MRI performed on a Siemens 1.5 T (Magnetom Avanto, Erlangen, Germany) system. T1-weighted axial and sagittal images in all subjects, fluid-attenuated inversion recovery axial images, and fluid-attenuated inversion recovery axial images (TR/TE/NEX/slice thickness = 9000–10,000/105–140/2/5–5.5) were obtained. Echo planar images were acquired with diffusion gradients at 1.5 T in x, y, and z planes using 5-mm-thick sections with 1.5 mm.

**Fig. 2:**
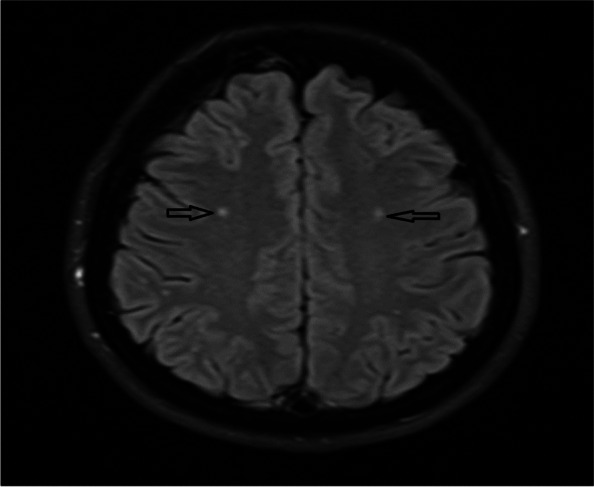
The white matter lesions in the cranial axial T2 (FLAIR) MRI in a patient with migraine (Black arrows showing the lesions)

The presence of high-signal-intensity punctate foci on T2WI and FLAIR images were regarded as the WMH. The subjects were separated into two groups: those with and without white matter hyperintense lesions (Fig. 2). WMH were considered if they were greater than three mm, and visible as hyperintense on T2-weighted and FLAIR images, without hypointensity on T1-weighted scans. A neuroradiologist reviewed all MRI studies for the involvement of the brain cerebral white matter by evaluation of the signal changes on T1 and T2-weighted sequences.

#### *Helicobacter pylori* detection

The endoscopy procedure was performed by an endoscopy specialist under midazolam and propofol sedation according to the age and characteristics of the patients. For bacteriological histopathological examination, biopsy specimens were taken from the antrum, corpus, and incisura. In the histological examination, all gastric biopsy specimens were fixed in 10% formalin and examined in the pathology department using Giemsa staining to detect *H. pylori* (plus hematoxylin and eosin preparation to assess gastritis lesions). The detection of *H. pylori* in the biopsy specimens was regarded as a positive result.

### Variables

Demographic information (age, sex), comorbidities (hypertension and diabetes mellitus (DM)), cranial MRI findings in terms of WML, and the results of *H. pylori* testing were recorded using the medical files of the patient.

### Statistical analyses

Statistical analyses were performed using SPSS for Windows vn. 22.0 software (SPSS Inc., Chicago, IL, USA). Categorical variables were expressed as number (n) and percentage (%) and continuous variables as mean ± standard deviation (SD) values. The one-sample Kolmogorov-Smirnov test was used to check the normality assumption of age and continuous variables. The Chi-square test, Fisher Exact test, and independent t-test compared the distributions of the categorical data between groups. For all tests, a value of *p* <  0.05 was considered statistically significant. Univariate and multivariate logistic regression analyses were performed to analyze the factors that impact the development of WML. Statistically significant and clinically essential factors in the univariate analysis were included in the multivariate analysis. Independent variables with multicollinearity problems were not included in the multivariate model.

## Results

The evaluation was made of a total of 526 migraine patients who met the inclusion criteria, comprising 397 (75.5%) females and 129 (24.5%) males with a mean age of 45.57 ± 13.46 years (range, 18–69 years). The mean age was 45.17 ± 13.288 years for females and 46.80 ± 13.955 years for males (Table 1).

**Table 1:** Participant demographic and clinical information

N (Patients) ^a^	WML		***p***-values
	WML (−) 277 (52.7)	WML (+) 249 (47.3)	
**Gender** ^a^			
Male	59 (45.7)	70 (54.3)	0.074
Female	218 (54.9)	179 (45.1)	
**Age** ^b^	40.30 ± 12.57	51.43 ± 11.93	0.010*
***H. pylori*** (+) ^a^	137 (26.0)	178 (33.8)	0.026*
**Diabetes mellitus** (+) ^a^	31 (5.9)	41 (7.8)	0.079
**Hypertension** (+) ^a^	60 (11.4)	112 (21.3)	0.001*

^a^: n (%), ^b^: mean ± standard deviation

*WML* White matter lesion, *H. pylori Helicobacter pylori*

* Mean difference is significant at the 0.05 level

**Fig. 3:**
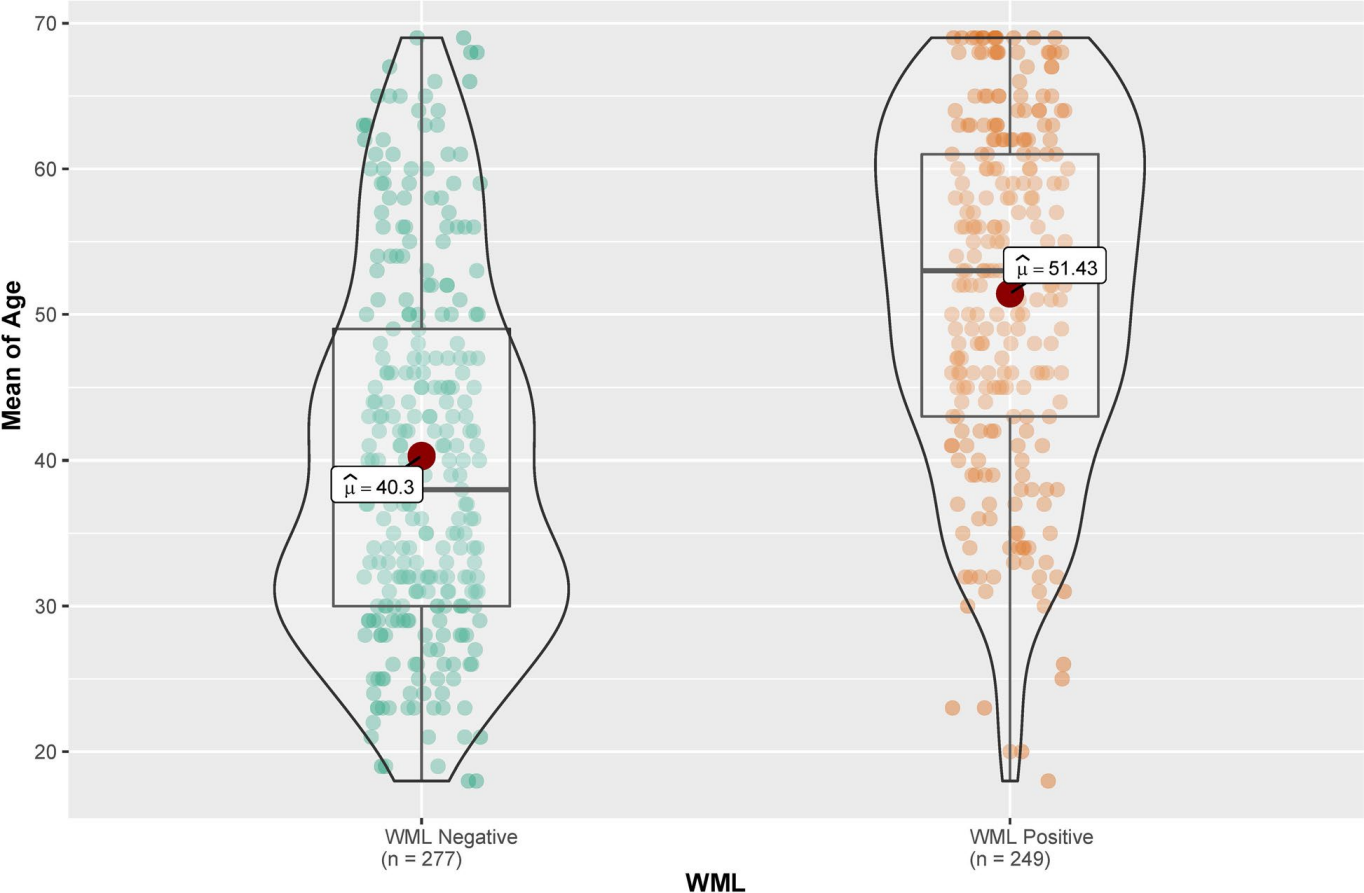
The Box-Violin graph shows the positive association between the rate of the white matter lesions and the age of the patients

The WML rates on brain MRI of patients with migraine are shown in Table 1. The age of the 277 (52.7%) WML negative migraine patients was 40.30 ± 12.56 years, and of the 249 (47.3%) WML positive patients was 51.43 ± 11.92 years (*p* <  0.05). As the patient’s age increased, the incidence of WML also increased (Fig. 3).

A total of 315 (59.8%) subjects with positive *H. pylori* were detected. Among those patients 178 (33.8%) of them were WML positive (*p* <  0.05) (Table 1). WML was 2.5-fold higher on brain MRI in patients with *H. pylori*-positive migraine (odds ratio: 2.562, 95% CI 1.784–3.680) (Table 2). WML was negative in 137 (26.0%) patients who were *H. pylori*-positive. WML was positive in 71 (13.5%) on brain MRI in patients who were negative for *H. pylori*.

**Table 2:** The univariate and multivariate logistic regression analyses of the factors affecting white matter lesion

	Univariate		Multiple	
	Crude OR [95%CI]	crude ***P*** value	Adjusted OR [95%CI]	adj. ***P*** value
**Age**	1.07 [1.06–1.09]	**< 0.001**	1.07 [0.01–0.04]	**< 0.001**
**Gender**: Male vs. female (ref.)	1.44 [0.97–2.15]	0.070	1.48 [0.94–2.34]	0.090
**HP**: Present vs. absent (ref.)	2.56 [1.78–3.68]	**< 0.001**	2.48 [1.65–3.72]	**< 0.001**
**Hypertension**: Present vs. absent (ref.)	2.96 [2.02–4.32]	**< 0.001**	4.52 [2.82–7.23]	**< 0.001**
**DM**: Present vs. absent (ref.)	1.56 [0.95–2.58]	0.080	2.01 [0.62–6.47]	0.241
DM*hypertension: Present vs. absent (ref.)	1.13 [0.63–2.00]	0.686	0.12 [0.03–0.45]	**0.002**

*Ref* Reference value, *H. pylori Helicobacter pylori*, *DM* Diabetes mellitus

In migraine patients with hypertension, WML was negative in 60 (11.4%) patients, and positive in 112 (21.3%) patients (*p* < 0.001). In migraine patients with diabetes mellitus, WML was not detected in 31 (5.9%) patients, and WML findings were found in 41 (7.8%) patients (*p* > 0.079).

There was no significant difference between DM and migraine patients with WML.

The univariate and multivariate logistic regression analyses were performed to evaluate the association between gender, *H. pylori*, hypertension, DM, and hypertension + DM in predicting WML development in migraine patients. The univariate analysis revealed that age, *H. pylori*, and hypertension were significantly associated with WML in this patient group (*p* < 0.05). The age (OR = 1.07, 95% CI: 0.01–0.04, *p* < 0.001), *H. pylori* (OR = 2.48, 95% CI: 1.65–3.72, *p* < 0.001), hypertension (OR = 4.52, 95% CI: 2.82–7.231, *p* < 0.001), and hypertension + DM (OR = 0.12, 95% CI: 0.03–0.54, *p* = 0.002), had significant associations in predicting WML according to the multivariate logistic regression analysis (Table 2). The presence of hypertension had a higher odds ratio value than the other variables.

## Discussions

Many risk factors have been defined for developing of WML in literature. In our study, we primarily aimed to determine the role of *H. pylori* status, in patients diagnosed with episodic migraine. Additionally, we also evaluated the patients’ demographic characteristics and if they were diagnosed with hypertension and/or DM. We detected that age, *H. pylori* and hypertension was significantly associated with WML in patients involved (*p* < 0.05). However, there were no significant relationship was present between gender, DM and WML.

Colonization of *H. pylori* in the stomach leads to humoral and cellular immune reactions that generally do not result in bacterial clearance [16]. The role of inflammation caused by *H. pylori* infection is still under debate in neurological conditions such as migraine, Guillain-Barré syndrome, multiple sclerosis, Parkinson’s disease, Alzheimer’s disease, and other inflammatory conditions such as ischemic stroke [23]. *H. pylori* infection results in a chronic inflammatory response with the local and systemic secretion of multiple inflammatory mediators. This response includes the chemokines, TNF-α, IL-8, macrophage chemotactic protein-1, and GRO [23, 24]. Studies investigating the relationship between *H. pylori* and migraine are ongoing [10, 13, 25].

WMH refers to lesions seen on MRI with ischemic features, which cause clinical signs or other stroke-related symptoms [26]. These lesions show an extensive dispersion in subcortical and deep white matter [27]. Incidentally, WMH on MRI increase in the population as they age and occur in up to 80% of individuals in the eighth decade, compared to about 10% of middle-aged individuals [28]. One study revealed that the prevalence of the WMH lesion in 65 migraine patients was 43.1% at varying degrees of hyperintensity [23]. In another study, a wide range of WMH frequency (4–71%) was reported for migraine patients [29]. The current study determined WML in 249 (47.3%) of 526 migraine patients, consistent with the literature.

Increasing age has been defined as a significant risk factor for WML [30, 31].

Consistent with previous studies, in our study, the rate of WML was seen to increase with age (*p* < 0.005).

WMH are commonly seen as multiple, small, dotted lesions in deep or periventricular white matter and are primarily observed in T2-weighted or FLAIR sequences [32]. Different hypotheses have been proposed regarding the mechanisms underlying WML in migraine patients. These are vascular risk factors (atherosclerosis, migraine, vasculitis, amyloid angiopathy) and non-vascular risk factors, such as cardiac abnormalities including inflammatory, metabolic, genetic, and neoplastic risk factors, infections, endothelial dysfunction, drugs, and the patent foramen ovale. Etiologically, infectious agents of WML include Herpes simplex virus, and Cytomegalovirus, encephalitis, neurosyphilis, central nervous system cryptococcal infection, progressive multifocal leukoencephalopathy, neuroborreliosis, human immunodeficiency virus encephalopathy, Whipple’s disease, Lyme encephalopathy, and subacute sclerosing panencephalitis [33–35]. In the present study, the effect of *H. pylori* infection, one of the risk factors for WML, was investigated in subjects with migraine. WML was detected on brain MRI at a rate 2.5-fold higher in H pylori-positive migraine patients (odds ratio: 2.562, 95% CI 1.784–3.680) (Table 1). This result was statistically significant. When previous studies on this subject were searched, no literature on the association between *H. pylori* and WML was found. In this respect, the current study can be considered as the first study on this subject.

Previous studies thought that these lesions are related to vascular risk factors and small vessel disease. This finding may be regarded as evidence for the ischemic origin. However, recent pathology studies have suggested a more complex pathophysiology [36]. In the multiple model regression analysis performed in the current study, in which risk factors related to the development of WML in migraine patients were evaluated collectively, *H. pylori*, age, hypertension and hypertension along with DM were seen to statistically significantly increase the occurrence of WML (*p* < 0.05).

The relationship between migraine and hypertension has been discussed for many years, and no definite conclusion has yet been reached as to whether there is a relationship. Hypertension and migraine are prevalent diseases in the general population, and their association suggests coincidental [37, 38]. Migraine can occur at a younger age and pose a risk for hypertension. In a prospective cohort study conducted in Finland, a self-reported physician diagnosis of migraine at baseline was related with an approximately 1.4-fold increased risk of diagnosing with hypertension after adjustments for age, gender, living alone, physical activity, body mass index, occupational training, and alcohol consumption [39]. In other studies, it has been suggested that increased systolic blood pressure predisposes migraine patients to the formation of white matter hyperintensities [29, 40]. In the current study, WML was more common in patients with coexisting migraine and hypertension than those without (*p* < 0.05). Therefore, a diagnosis of hypertension with the increasing age of the patient can be said to be a risk factor for the development of WML.

WML has been considered to indicate injury of small vessels in the periventricular and subcortical areas, and autopsy series have indicated that the pathogenesis of WML consists of myelin pallor, tissue myelin rarefaction, and enlargement of perivascular spaces [41]. However, evidence that DM and WML may be related is inconsistent [42, 43]. The co-existence of atrophy with greater WML volume suggests that type 2 DM may be related to mixed pathology in the brain [44]. In our study, no significant difference was determined regarding the WML detection rate in patients with DM and migraine (*p* > 0.05). Factors that advance the formation of WML in older subjects with diabetes have not been fully defined. As the consequences of DM, hypertension, dyslipidemia or other possible vascular risk factors have not been fully defined. There is a need for further studies.

The data in the current study indicated that when hypertension and DM were evaluated together, the WML rate was significantly increased in subjects with migraine (*p* > 0.05).

The retrospective design was the major limitation of the study. In addition to the retrospective design, considering that many patients may had been misdiagnosed or followed up in other clinics, we were not able to determine the effect of the duration of a patient suffering from migraine. Their medication histories were also not recorded clearly. The possible association between migraine and *H. pylori* infection is a complex subject leading to difficulties for a retrospective study. Our study did not evaluate the impact of several metabolic and endocrine diseases causing white matter lesions in the brain. The absence of this data should be considered.

In conclusion, in most neurological diseases, WML occurs due to the combination of multiple factors. The results of this study indicated that one of these factors could be *H. pylori*, as the presence of *H. pylori* was seen to increase the development of WML in subjects with migraine. The increase in comorbid conditions and age with *H. pylori* further increased the incidence of WML. Therefore, it was concluded that *H. pylori* infection, as a chronic infection, can be counted among the risk factors for the development of WML in migraine patients. Thus, it can be considered that *H. pylori* eradication will be essential to reduce the development of WML in subjects with migraine and consequently reduce the risk of morbid conditions such as stroke due to WML. Nevertheless, further prospective studies are needed for a better understanding of this issue.

## Supplementary Information

**Additional file 1.**

## Data Availability

The data used within this article will be made available.
